# Supplementary material for: Content-rich biological network constructed by mining PubMed abstracts
Source: BMC Bioinformatics. 2004 Oct 8;5:147. doi: 10.1186/1471-2105-5-147 (PMC528731; doi:10.1186/1471-2105-5-147)
Supplement: Additional File 2 — The original results of the above study (non-essential files are deleted to keep the file size under the limit set by BMC bioinformatics). [file 1471-2105-5-147-S2.bz2 › chilibotAdditionalFile2/dip10/149ID9171381E537/html/VIRB7_VIRB9.html]

 


 **VIRB7** and **VIRB9** 
  
Found 20 abstracts in PubMed, retrieved 10.  
 

 What does Google say? 
 PDF only 
| .edu only 

---

**Interactive relationship** (e.g. stimulation, inhibition, etc)

**Neutral relationship**- We established interactions between VirB11 an inner membrane pore forming protein,  **VirB9**  a periplasmic protein, and  **VirB7**  an outer membrane associated lipoprotein and putative pilus component.  Ref: 12177441 Proc Natl Acad Sci U S A, 2002
- **VirB7**  assembles as a disulfide cross linked homodimer that associates with the T pilus and a  **VirB7**   **VirB9**  heterodimer that stabilizes other VirB proteins during biogenesis of the secretion machine.  Ref: 12700266 J Bacteriol, 2003

**Non-interactive relationship** (e.g. studied together, co-existance, homology, etc.)

- Fractionation of  **VirB7**  containing complexes  **VirB7**   **VirB7**  homodimers and  **VirB7**   **VirB9**  heterodimers suggested that they may link the T pilus components to the core of the translocation machinery.  Ref: 12177443 Proc Natl Acad Sci U S A, 2002
- Strains producing several VirB6.i4 mutant proteins assembled novel  **VirB7**  and  **VirB9**  complexes detectable by nonreducing sodium dodecyl sulfate polyacrylamide gel electrophoresis, and two strains producing the D60.i4 and L191.i4 mutant proteins translocated IncQ plasmid and VirE2 effector protein substrates in the absence of a detectable T pilus.  Ref: 12700266 J Bacteriol, 2003
- The second class contains putative translocation complex core components VirB8,  **VirB9** , and VirB10 in the high molecular mass portion of the gel larger than 232 kDa, as well as  **VirB7** .  Ref: 12177443 Proc Natl Acad Sci U S A, 2002
- Finally, the membrane distribution and cross linking patterns of VirB10, but not of VirB8 or  **VirB9** , in a strain expressing only  **VirB7**  to VirB10 are significantly altered compared to the patterns of the wild type.  Ref: 12754223 J Bacteriol, 2003
- To examine the biological importance of VirB6 complex formation for type IV secretion, we monitored the effects of nonstoichiometric VirB6 production and the synthesis of VirB6 derivatives with 4 residue insertions VirB6.i4 on  **VirB7**  and  **VirB9**  multimerization, T pilus assembly, and substrate transfer.  Ref: 12700266 J Bacteriol, 2003
- In the absence of VirB6, cell bound  **VirB7**  monomers and  **VirB7**   **VirB9**  heterodimers were reduced and  **VirB7**  homodimer formation was abolished.  Ref: 10913084 J Bacteriol, 2000
- Our findings support a model that VirB6 mediates formation of  **VirB7**  and  **VirB9**  complexes required for biogenesis of the T pilus and the secretion channel.  Ref: 12700266 J Bacteriol, 2003
- Furthermore, the filamentous structures on bacterium were found to be associated with Hp0532  **VirB7**  or Hp0528  **VirB9** , the major components of TFSS, by immunogold electron microscopy.  Ref: 12780777 Cell Microbiol< MedlineTA>Cell Microbiol, 2003
- These results suggest that the outer membrane  **VirB7**   **VirB9**  complex interacts with the inner membrane proteins VirB8 and VirB10 for the assembly of the transport pore.  Ref: 10633111 J Bacteriol, 2000
- We studied interaction among four putative transport pore proteins,  **VirB7** , VirB8,  **VirB9**  and VirB10.  Ref: 10633111 J Bacteriol, 2000
- Whereas class I proteins VirB2,  **VirB7** ,  **VirB9** , and VirB10 were readily detected at 28 degrees C, class II proteins VirB1, VirB4, VirB5, VirB6, VirB8, VirB11, VirD2, and VirE2 were only detected after cell growth below 26 degrees C.  Ref: 11698374 J Bacteriol, 2001
- Here, we analyzed the function of 12 cag PAI genes from both cag I and cag II loci, including the complete virB D complex virB4,  **virB7** , virB8,  **virB9** , virB10, virB11, and virD4.  Ref: 11796597 Infect Immun, 2002
- This study characterized the contribution of Agrobacterium tumefaciens VirB6, a polytopic inner membrane protein, to the formation of outer membrane  **VirB7**  lipoprotein and  **VirB9**  protein multimers required for type IV secretion.  Ref: 12700266 J Bacteriol, 2003
- Agrobacterium tumefaciens VirB6 protein participates in formation of  **VirB7**  and  **VirB9**  complexes required for type IV secretion.  Ref: 12700266 J Bacteriol, 2003
- Two presumptive VirB protein complexes, composed of VirB6,  **VirB7** , and  **VirB9**  and of  **VirB7** ,  **VirB9** , and VirB10, were isolated by immunoprecipitation or glutathione S transferase pulldown assays from detergent solubilized membrane extracts of wild type A348 and a strain producing only VirB6 through VirB10 among the VirB proteins.  Ref: 12700266 J Bacteriol, 2003
